# Local Excision ± Chemoradiotherapy vs. Total Mesorectal Excision for Early Rectal Cancer: Case-Matched Analysis of Long-Term Results

**DOI:** 10.3389/fsurg.2021.746784

**Published:** 2021-10-18

**Authors:** Julius Pacevicius, Vidas Petrauskas, Lukas Pilipavicius, Audrius Dulskas

**Affiliations:** Department of Abdominal and General Surgery and Oncology, National Cancer Institute, Vilnius, Lithuania

**Keywords:** early rectal cancer, local excision, total mesorectal excision, chemoradiotherapy, survival, functional outcome

## Abstract

**Background:** Our aim was to compare the bowel function and oncologic outcomes following these two treatment modalities.

**Materials and methods:** This was a single-center study with 67 patients included between 2009 and 2018. A total of 32 patients underwent total mesorectal excision (TME) group and 35 transanal local excisions (LE) ± chemoradiation. We performed a case-matched analysis: we matched the patients by age, cancer stage, and comorbidities. Duration of operation, postoperative complications, length of hospital stay, and long-term functional and oncological outcomes were compared. We calculated oncological outcomes using *Kaplan–Meier* Cox diagrams. In addition, we used a low anterior resection syndrome (LARS) score for the bowel function assessment.

**Results:** Mean operation time in the LE group was 58.8 ± 45 min compared with the TME group that was 121.1 ± 42 min (*p* = 0.032). Complications were seen in 5.7% in LE group and 15.62% in TME group (*p* = 0.043). ~85.2% of the patients had no LARS in LE group compared with 54.5% in TME group (*p* = 0.018). Minor LARS was 7.4% in LE group compared with 31.8% in TME group (*p* = 0.018); major LARS was 7.4 and 13.7%, respectively (*p* = 0.474). Hospital stay was 2.77 days in LE group compared with 9.21 days in TME group (*p* = 0.036). The overall survival was 68.78 months in LE group compared with 74.81 months in TME group (*p* = 0.964).

**Conclusion:** Our results of a small sample size showed that local excision ± chemoradiation is a rather safe method for early rectal cancer compared with gold standard treatment. In addition, better bowel function is preserved with less postoperative complications and shorter hospital stays.

## Introduction

Colorectal cancer is common cancer worldwide with rectal cancer accounting for approximately 30% of all colorectal malignancies (1). Due to its location and dissemination, treatment of rectal cancer remains challenging. Over the last three decades, the gold standard treatment was total mesorectal excision (TME) with or without neoadjuvant chemoradiotherapy, which has shown significant improvements with respect to local disease control (2). However, this treatment is associated with certain numbers of mortality (4%) and morbidity (from 6 to 35%) (3, 4). Up to 75% of these patients eventually will experience bowel, urogenital dysfunction seriously affecting the quality of life (5).

Recently, thanks to cancer screening programs, the proportion of rectal cancers diagnosed at an early stage are increasing in Western countries, which gives the capability of reducing the size of the operation and minimizing negative effects on low anterior resection with TME (6). Now, minimally invasive local excision (LE) techniques, in addition to standard transanal excision (TE) with chemoradiation, can be used as an alternative to radical excision (7). LE plus chemoradiotherapy approach possibly decreases the risk of bowel dysfunction and gives acceptable local/distant recurrence rates by decontaminating the mesorectal lymph nodes and the excision bed. It is later translated to lower morbidity and comparable long-term survival results (7–9). Nevertheless, there is limited knowledge on the long-term functional and oncological results of TME vs. LE ± chemoradiotherapy for early rectal cancer.

We aimed to compare the long-term bowel function and oncologic outcomes following these two treatment modalities.

## Methods

### Patients and Groups

The National Cancer Institute Review Board has approved the study (approval number NCI 2019.129AK). All the patients signed the written informed consent.

**Figure 1:**
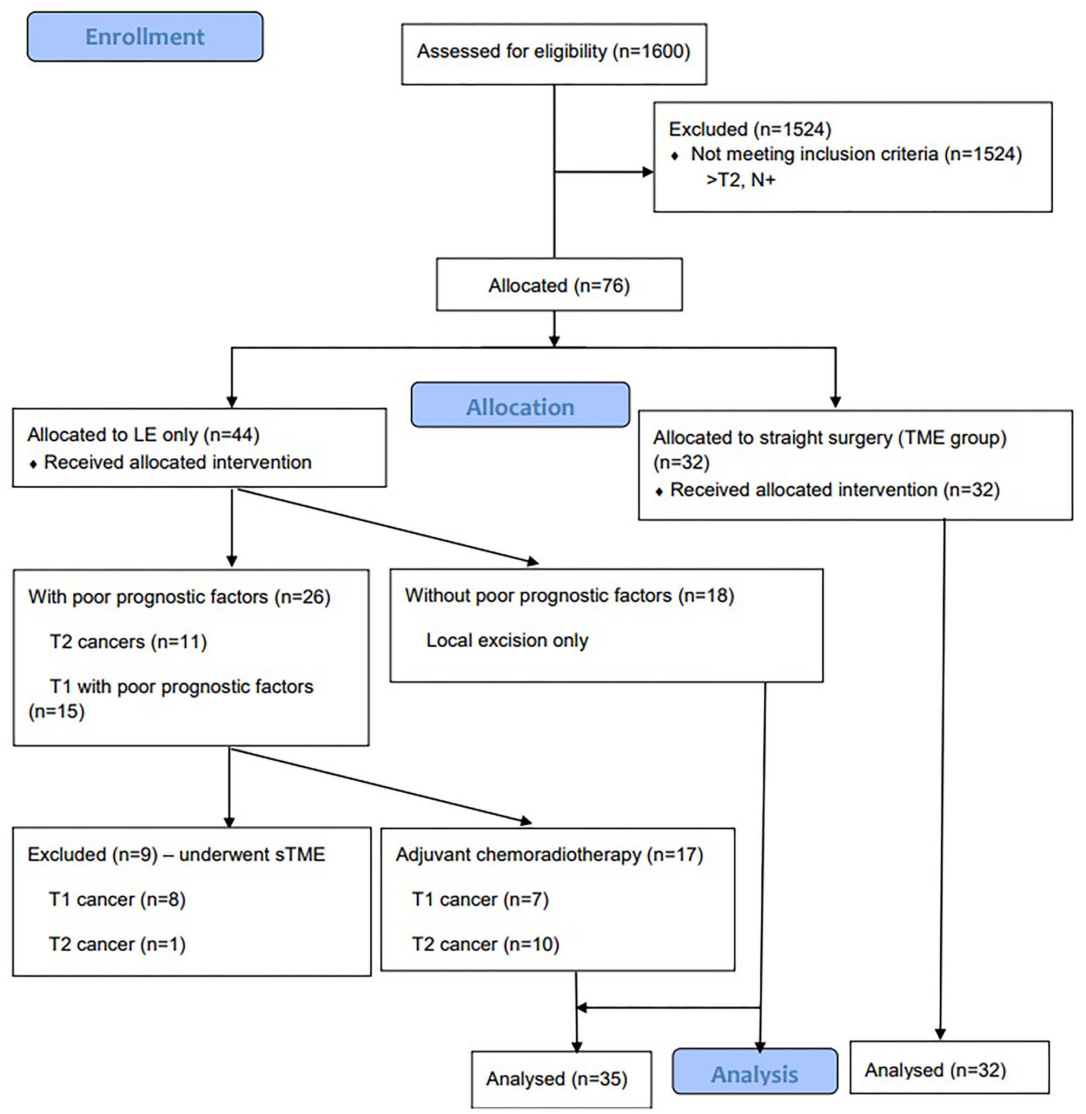
Consort Flow Diagram of patients undergoing local excision with or without chemoradiotherapy vs total mesorectal excision for early rectal cancer.

Data from the consecutively recruited patients who were treated at the National Cancer Institute between 2009 and 2018 were investigated. Patients who had T1-T2 rectal cancer with no lymph node or distant metastases (staging was done by using CT scan of the chest and abdomen and MRI of the pelvis) and with final pathology were included. We excluded patients with more than pT2 cancers and patients with positive lymph nodes (either on staging MRI or on final pathology). During the study period, more than 1,600 rectal cancer surgeries were performed (see in Figure 1). All the surgeries were performed by the five surgeons with experience of at least 5 years. The type of operation was determined by considering age of the patient, comorbidities, preference of the patient, and the size of the tumor. In total, there were 67 cases: 32 cases with TME group and 35 cases with transanal LE ± chemoradiation – LE group. All the patients in the TME group underwent straight radical open surgery with stapled coloanal anastomosis without previous LE techniques. Patients in the LE group underwent either transanal endoscopic microsurgery (TEM) or transphincteric excision. We matched both groups by age, cancer stage, and comorbidities. The mean follow-up duration of the patients was more than 3 years. Patients every 3 months for 2 years underwent carcinoembryonic antigen (CEA) evaluation, chest X-ray, ultrasound of the abdomen, or CT scan of the abdomen/chest, later every 6 months and then once a year. A mass in the pelvis around or in anastomosis site found by clinical, endoscopic, radiologic, pathologic examination, or autopsy was defined as local recurrence (or in pelvic lymph nodes in cases when LE was performed). Similarly, distant recurrence was defined as tumor growth in any lymph node outside the pelvis or in any other organ.

We used a low anterior resection syndrome (LARS) score for bowel function assessment for at least 2 years following the procedure (10, 11). Complications were graded by using *Clavien–Dindo* classification (12).

If the final pathology report following local excision was T2 cancer (10 patients) or T1 cancer with poor prognostic factors (seven patients) (such as positive margin, lymphovascular invasion, poor differentiation—G3 and Sm3), the patient was offered completion of TME or adjuvant chemoradiotherapy, if the patient was unfit or unwilling for the surgery. Patients received 50.4–54.0 Gy of radiation to the pelvis concomitant to 5-fluorouracil-based chemotherapy for 5 weeks (1.8–2 Gy per day). Seventeen patients underwent chemoradiotherapy. We have excluded nine patients who had poor prognostic factors and underwent completion of TME (13).

We have also performed a subgroup analysis and compared the survival and bowel function in three groups: LE only, LE + chemoradiotherapy, and TME group.

### Statistical Analysis

We performed statistical analysis using SPSS Statistics 23.0 (IBM Corporation, released 2015, IBM SPSS Statistics for Windows, Version 23.0. Armonk, New York). The *Kaplan–Meier* Cox diagrams were calculated for oncologic outcomes.

The sample size was calculated by using G^*^Power 3.1.9.4 sample size calculator and the free version was available from https://stats.idre.ucla.edu/other/gpower/ (accessed on August 31, 2021). The value of alpha—the probability of a false positive was set at 5% and, hence, the familiar *p* < 0.05. Power is 1-beta, so in percentage terms, these were expressed as 80%. The effect size was set at 0.15 (the expected difference of patients having major LARS between the two groups of 15%). For 1:1 randomization, it showed that 44 patients (22 in each arm) would provide 80% power for a two-sample proportion test. There are likely to be patients lost to follow-up, so the target recruitment was set at 50.

## Results

The demographics of the patient are highlighted in Table 1. The mean duration of operation in the LE group was 58.8 ± 45 min compared with the TME group that was 121.1 ± 42 min (*p* = 0.032). Two patients (5.7%) in the LE group had complications: one patient was treated conservatively, one had grade IIIB complication—fistula, which required additional surgical intervention and five patients in TME group (15.62%) (*p* = 0.043) had grade II-IIIA complications. The length of hospital stay in LE group was 2.77 days and 9.21 days in the TME group (Table 2). In the LE group, 17 (49%) patients received adjuvant chemoradiotherapy.

**Table 1:** Patient and tumor characteristics of both the study groups.

**Category, data (*n* = 67)**	**Groups**	
	**LE (*n* = 35)**	**TME (*n* = 32)**
Age range (average), years	69 ± 11 (from 51 to 88)	66 ± 8 (from 45 to 75)
Sex, *n* (%) •Male (*n* = 45) •Female (*n* = 22)	23 (51.11%) 12 (54.54%)	22 (48.89%)10 (45.46%)
T stage, *n* (%) •T1 (*n* = 47) •T2 (*n* = 20)	25 (53.19%) 10 (50%)	22 (46.81%)10 (50%)
Tumor height from anus, *n* (%) • <6 cm (*n* = 25) •6–12 cm (*n* = 42)	17 (68%) 18 (43%)	8 (32%)24 (57%)

*LE, local excision; TME, total mesorectal excision*.

**Table 2:** Comparison of two groups included in our study (LE, local excision group and TME, radical surgery group).

**Groups**	**LE**	**TME**	***p*-value**
Patient number, *n* (%)	35 (52%)	32 (48%)	
Operating time (average), min	58.8 ± 45(from 15 to 300)	121.1 ± 42(from 45 to 225)	0.032
Complications, *n* (%)	2 (5.7%)	5 (15.62%)	0.043
Hospital stay, days	2.77 ± 2.5 (from 1 to 15)	9.21 ± 4.2 (from 5 to 14)	0.036
Oncological recurrence, *n* (%)	1 (2.9%)	0 (0%)	
Survival, months	68.78	74.81	0.964
Follow-up, months	34 ± 21 (from 25 to 82)	37 ± 20 (from 24 to 85)	0.870

*TME, total mesorectal excision*.

In LE group, out of 35 patients, 25 patients (71.4%) underwent TME.

**Figure 2:**
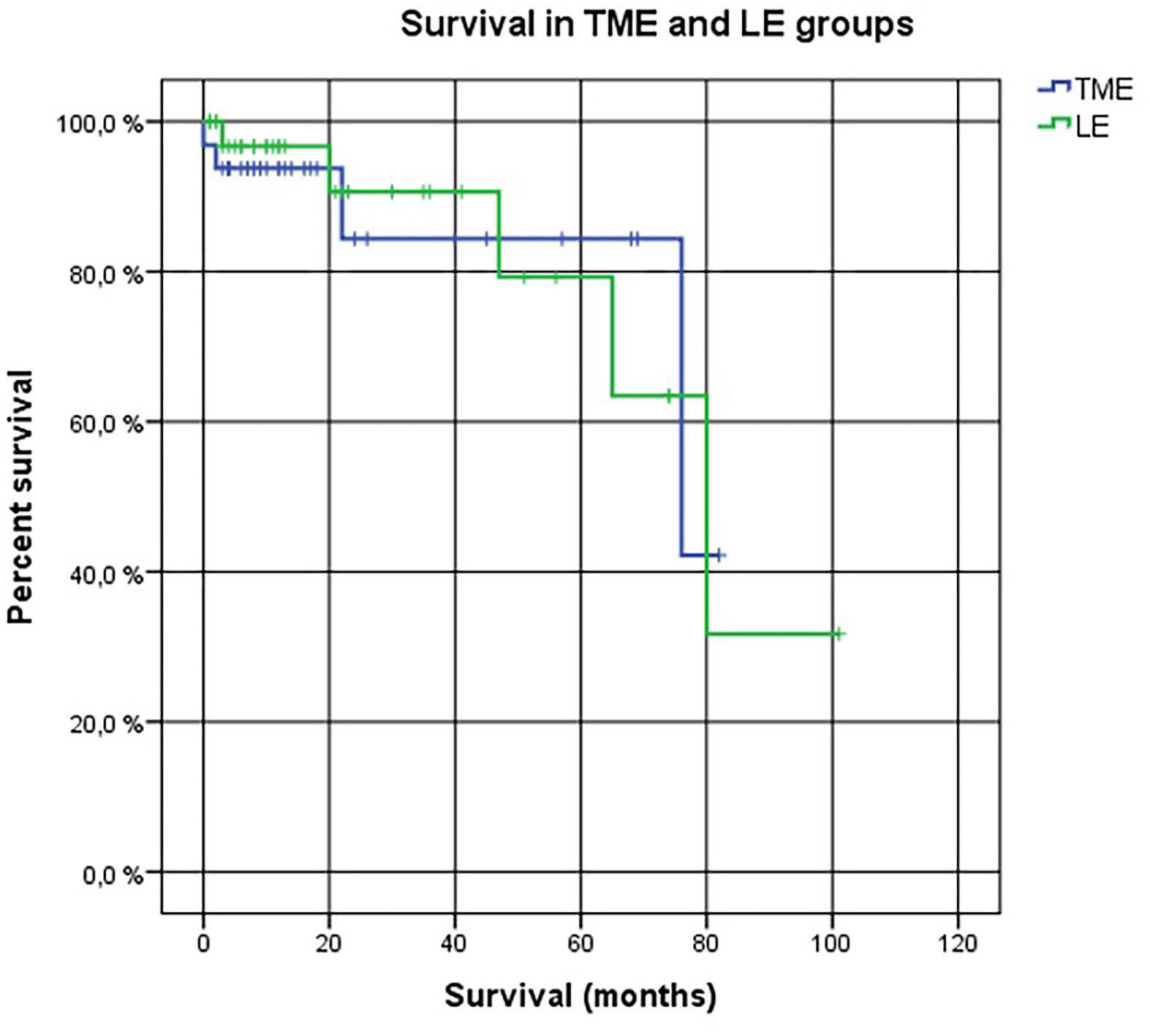
The *Kaplan–Meier* Cox diagrams for evaluating survival in two groups. Log Rank (Mantel-Cox) *p*-value = 0.964. LE, local excision. TME, total mesorectal excision.

No LARS was found in 85.2% of the patients in LE group compared to 54.5% of the patients in the TME group (*p* = 0.018). Minor LARS was 7.4% in LE group compared to 31.8% in TME group (*p* = 0.018); major LARS was 7.4 and 13.7%, respectively (*p* = 0.474) (Table 3). There was no statistically significant difference in overall survival between the two groups: 68.78 months in the LE group and 74.81 months in the TME group (*p* = 0.964) (Figure 2). Local recurrence was detected in one (2.9%) patient in the LE group 6 years following the treatment compared to the TME group 0 year. The patient underwent abdominoperineal excision with a final pT3N0 pathology.

**Table 3:** Low anterior resection syndrome (LARS) comparison between the two groups.

**Groups**	**LE (*n* = 27)**	**TME (*n* = 22)**	***p*-value**
No LARS, *n* (%)	23 (85.2%)	12 (54.5%)	0.018
Minor LARS, *n* (%)	2 (7.4%)	7 (31.8%)	0.028
Major LARS, *n* (%)	2 (7.4%)	3 (13.7%)	0.474
LARS	4 (14.8%)	10 (45.5%)	0.043

*LE, local excision; TME, total mesorectal excision*.

**Figure 3:**
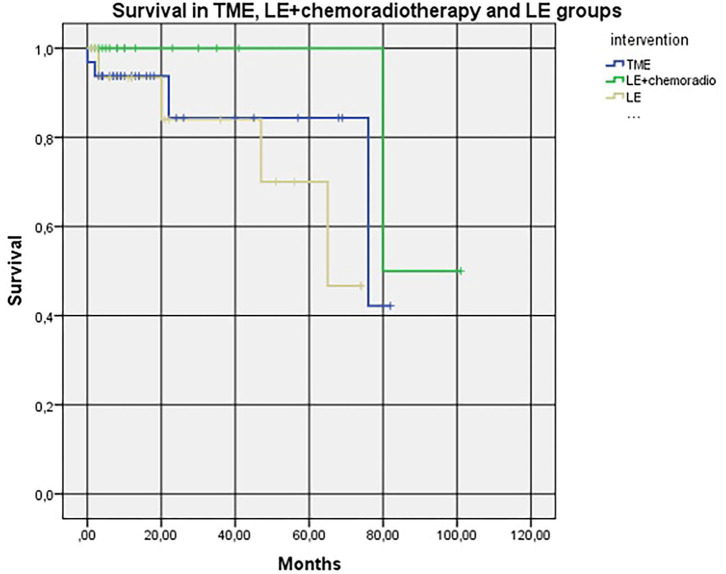
*Kaplan–Meier* Cox diagrams evaluating survival in three groups (subgroup analysis). Log Rank (Mantel-Cox) *p*-value = 0.236. LE+chemoradio-local excision + chemoradiotheraphy. LE, local excision. TME, total mesorectal excision.

In addition, in a subgroup analysis, we found no LARS in 12 (54.5%) patients who underwent TME, in 12 (92.3%) patients with LE ± chemoradiation, and in 11 (78.6%) patients with LE only (*p* = 0.045). Accordingly, major LARS was present in three (13.6%) patients, one (7.7%) patient, and one (7.1%) patient (*p* = 0.7330). Moreover, we found no survival difference between the three groups (*p* = 0.236) (Figure 3).

In both groups, 13 patients (seven patients in the LE group and six patients in the TME group) had poor prognostic factors. However, as the numbers are very small, no further analysis was performed.

## Discussion

We found that LE with or without chemoradiation can provide good oncological and functional outcomes compared with radical surgery (TME). LE remains an evolving area in the management of rectal cancer, requiring comprehensive screening and selection of patients. Nevertheless, the right choice of treatment can significantly improve quality of life of the patient without compromising survival. However, LE for high-risk T1 or T2 rectal carcinomas is a relative contraindication because it is associated with a high risk of local or distant recurrence compared to radical surgery (7, 14).

Cancer recurrence is one of the most important indicators when talking about alternative treatment modalities compared to gold standard treatment—TME. In a large meta-analysis, *Borstlap et al*. found the overall local recurrence after LE following adjuvant chemoradiotherapy that was 5% for pT1 rectal cancer and 14.3% for pT2 rectal cancer. Distant recurrence for pT1/pT2 rectal cancer was 8.2% (7). Furthermore, a large Norwegian national observational study including more than 2,000 patients showed that transanal endoscopic microsurgery (TEM) had similar 5-year survival rates to the TME group in T1 rectal cancer, but lower 5-year relative survival in T2 rectal cancer. TEM also had higher local recurrence rates for T1 and T2 cancers (15). In a recent systematic review by *You et al*. including about 800 patients, the local recurrence rate after LE ± chemoradiotherapy was 5.8% for pT1, 13.8% for pT2, and 33.7% for pT3 tumors (16). Some studies show that the recurrence rate is relatively higher after LE alone compared with TME (15, 17, 18).

In this study, not all the patients underwent chemoradiotherapy after surgery, so it raises a question—how chemoradiotherapy additionally affects oncological outcomes. *Cutting et al*. in their systematic review draw attention that the evidence addressing the outcomes of the patients receiving adjuvant therapy after LE is lacking. Despite these limitations, the patients following LE and adjuvant treatment for high-risk early rectal cancer can sustain an acceptable long-term outcome (16). Documented data suggest that LE for pT1 tumor can recur locally in 8.2 to 23% and in pT2 tumor up to 30% (19). Our study results are corresponding to those mentioned above with 2.9%—although we observed a better recurrence rate, it must be considered that we had a smaller amount of the patients. Other authors suggest that in T1 rectal cancer, LE with additional chemoradiotherapy gives sufficient local control making it an acceptable treatment possibility in unfit patients or refusing radical surgery (20). *Rackley et al*. showed that early-stage cancer additionally affected with chemoradiotherapy has a 5-year local control of 92.5% (84.3–100%) for T1 cancer and 78.2% (65.5–90.9%) for T2 cancer. In addition, they stated that the LE and chemoradiotherapy were not recommended to be used in advanced disease (high-risk T2/T3 cancer). Interestingly, they found no local recurrence in the patients with T3 cancer. It is possible because these patients were typically very friable and died even before the development of recurrence with a 5-year overall survival rate of just 20 (21).

It is important to recognize that chemoradiotherapy is not so harmless. It is known that pelvic organ function worsens the following chemoradiotherapy with surgery compared to those who underwent surgery alone (22). Chemoradiotherapy has a significant negative effect and may lead to a spectrum of acute and late toxicities such as ulceration, bleeding, diarrhea, or problems of the skin. According to literature, 30–40% of the patients had chronic diarrhea, about 15% of the patients had obstructions, and even half of the patients had anorectal dysfunction after chemoradiotherapy (23–25). However, chemoradiotherapy is improving in areas warranting future research, such as advanced chemoradiation delivery techniques and risk-stratified patient management approaches are evolving and hopefully, it will cause a less negative effect in the future.

Furthermore, the importance of the quality of life of the patient after surgery should also be taken into consideration because often intervention has a negative impact on long-term bowel function and urogenital function. A study by *Pucciarelli et al*. states that when bowel function and quality of life after LE and TME were compared, LE revealed better results in all the bowel functions such as increased stool frequency (LE−12.8% vs. TME−25.8%), developed fecal incontinence (LE−9.9% vs. TME−24.8%), pain (LE−3.6% vs. TME−15.3%), and impotence (LE−33.3% vs. TME−62.3%) (26). Similar to these results, in our study, we found that LARS occurred in 14.8% of the patients in the LE group vs. 45.5% of the patients in the TME group. As already mentioned before, radiotherapy has a considerable negative effect—not only causes the development of complications but generally also affects anorectal function. Therefore, the need to evaluate LARS score occurs—several randomized controlled trials have demonstrated an almost 2-fold higher LARS prevalence in patients undergoing chemoradiotherapy with surgery compared to surgery alone (27, 28). In a recent study by *Ihnát et al*., authors compared LARS score following the surgery with or without radiotherapy and found that in the surgery alone group, 14.8% of the patients had major LARS and 37.0% of the patients had minor LARS compared to surgery plus radiotherapy group−53.6% of the patients with major LARS and 31.6% of the patients with minor LARS (29). In this study, the effect of chemoradiotherapy was not investigated, which could be added in future research.

Recently, the issue of treatment of early rectal cancer brought even more attention. Two systematic reviews and meta-analyses have been just published (30, 31). In both, the authors concluded that LE is safe for the treatment of early rectal cancer (this is T1 without poor prognostic factors). For T1 cancer with poor prognostic factors, chemoradiotherapy is a possible alternative to surgery and for T2 cancer—completion of TME should be the standard of care. This is in line with our results. However, because of a relatively small number of cases, we could not show the benefit of surgery in T2 cancers. Moreover, a group of experts from the STAR-TREC trial proposed limited irradiation volume for early rectal cancer to reduce toxicity and pelvic organ dysfunction (32, 33). However, this is the only theoretical proposal and the results of this trial should be awaited.

Our study is limited by the small sample size and retrospective approach. However, previous studies had very similar numbers of included patients. Moreover, in the LE group, there might have been more fragile older patients with the inability to survive the radical surgery. In addition, the follow-up of our last patients included is only 3 years—this weakens our statement on equal survival rates. As only one patient within the surveillance period had local recurrence, counting disease-free survival or local recurrence-free survival becomes irrelevant. Finally, the lack of endoanal ultrasound for preoperative examination is another limitation. The strength of our study is the assessment of bowel function in two selected groups by using a validated questionnaire.

## Conclusion

According to our small group, LE ± chemoradiation probably gives comparable results to TME in survival rates. On the contrary, it has better bowel function, causes fewer postoperative complications, and helps to shorten the length of hospital stay. However, patients with T2 cancer should be warned of the high risk of recurrence.

## Data Availability Statement

The data are available upon reasonable request from the corresponding author.

## Ethics Statement

The studies involving human participants were reviewed and approved by National Cancer Institute review board. The patients/participants provided their written informed consent to participate in this study.

## Author Contributions

AD, JP, and LP contributed to the study design and edition, patient enrollment, follow-up, data collection, final statistical analysis, text creation, and final text supervision. VP contributed with statistical analysis, text creation, and final text supervision. All authors listed have made a substantial, direct, intellectual contribution to the work, and approved it for publication.

## Conflict of Interest

The authors declare that the research was conducted in the absence of any commercial or financial relationships that could be construed as a potential conflict of interest.

## Publisher's Note

All claims expressed in this article are solely those of the authors and do not necessarily represent those of their affiliated organizations, or those of the publisher, the editors and the reviewers. Any product that may be evaluated in this article, or claim that may be made by its manufacturer, is not guaranteed or endorsed by the publisher.
